# Diagnosis and Management of Dengue Fever in a Nonendemic Country: Lessons From an Acute Febrile Illness in Iran During the COVID-19 Outbreak

**DOI:** 10.1155/crdi/5742576

**Published:** 2025-02-23

**Authors:** Kimia Mozahheb Yousefi, Solaleh Aminian, Masoud Ebrahimi, Sara Minaeian, Azadeh Laali

**Affiliations:** ^1^Antimicrobial Resistance Research Center, Institute of Immunology and Infectious Diseases, Iran University of Medical Sciences, Tehran, Iran; ^2^Student Research Committee, School of Medicine, Iran University of Medical Sciences, Tehran, Iran; ^3^Department of Infectious Disease, Shahid Modarres Hospital, School of Medicine, Shahid Beheshti University of Medical Sciences, Tehran, Iran; ^4^Department of Infectious Disease, Firoozgar General Hospital, School of Medicine, Iran University of Medical Sciences, Tehran, Iran; ^5^Faculty of Medicine, Iran University of Medical Sciences, Tehran, Iran

**Keywords:** coinfection, COVID-19, dengue fever, fever of unknown origin, nonendemic

## Abstract

Dengue fever is a tropical arboviral disease that presents with a broad spectrum of clinical manifestations and can occasionally emerge in nonendemic regions due to factors such as international travel. This case report details a 47-year-old Iranian man who had recently returned from Jakarta, Indonesia, presenting with fever, diarrhea, vomiting, and myalgia during the COVID-19 outbreak. Despite initial misdiagnoses, dengue fever was confirmed through polymerase chain reaction (PCR) testing. Moreover, the serological analysis using enzyme-linked immunosorbent assay (ELISA) further demonstrated the presence of both IgM and IgG antibodies against the dengue virus. Initially, the patient's symptoms overlapped with COVID-19 and gastrointestinal infections, complicating the diagnosis. The management included supportive care, precautions against bleeding, fluid therapy, and empirical antibiotics due to suspected coinfections. This case highlights the importance of considering travel history and the possibility of nonendemic diseases in the differential diagnosis of febrile illnesses of unknown origin (FUO), particularly during concurrent outbreaks. Comprehensive history-taking and rigorous diagnostic evaluation are essential in such cases.

## 1. Introduction

Dengue is an acute febrile disease caused by the dengue virus (DENV) and transmitted by female Aedes mosquitoes (Ae. aegypti and Ae. albopictus), frequently occurring in tropical and subtropical regions such as the Caribbean, Central and South America, Southeast Asia, and the Pacific Islands [1, 2]. As of the beginning of 2024, over 13 million cases of dengue and more than 8500 fatalities attributed to the disease have documented across 84 countries and territories, highlighting the ongoing global burden of this disease [3]. Serious presentations of dengue such as dengue hemorrhagic fever (DHF) and dengue shock syndrome (DSS) can lead to death due to plasma leakage, hypovolemic shock, and multiorgan failure [4].

While this disease is typically prevalent in endemic regions, it can also emerge in nonendemic areas due to various factors, including travel, climate change and urbanization, vector transmission, and newly recognized routes of transmission such as blood transfusion [5]. Because dengue is uncommon in nonendemic areas and presents with a wide range of nonspecific symptoms, it can mimic other febrile hemorrhagic diseases, posing challenges for healthcare providers who may misidentify it for other illnesses. This can result in missed diagnoses and potentially fatal consequences for patients.

The Middle East is generally considered a nonendemic area for dengue [6, 7]. Like other nonendemic countries in the region, Iran has not experienced many cases of dengue fever, with the first reported case occurring in 2008 [8]. In this article, we reported a confirmed case of dengue in Iran during the 2022 COVID-19 outbreak, which was initially misdiagnosed as other febrile diseases before being identified. To the best of our knowledge, this is the first reported case of dengue during the COVID-19 outbreak in Iran. This scenario not only brings attention to new dengue cases in the area and the emerging trend but also underscores the importance of considering nonendemic diseases, highlighting the diagnostic challenges, especially during the simultaneous prevalence of other febrile illnesses such as COVID-19, and exploring the epidemiological landscape of dengue in the Middle East and its intersection with other infectious diseases.

## 2. Case Presentation

The presented case is a 47-year-old Iranian man, who served as a referee for an international wushu tournament in Jakarta during his 10-day visit. He was admitted to the emergency room of Firoozgar Hospital in Tehran on December 12th, 2022, two day after his return to Iran. Upon his arrival to Iran, he presented with symptoms including fever, nausea, frequent vomiting, and oral intolerance. He had subsequently visited several physicians and hospitals and been tested for COVID-19, which yielded a negative result. Four days before his admission, he had sought the advice of the team physician in Jakarta due to the onset of diarrhea and lower abdominal cramps and was prescribed omeprazole with a potential diagnosis of gastroenteritis. Over the next two days, his symptoms escalated, with fever, chills, sweating, increased fatigue, frontal headache, ostealgia, myalgia, and arthralgia.

Based on the history, the vomit contained undigested food particles, and it was neither bilious nor mixed with blood. His fever was persistent throughout the entire day, had no specific pattern, and improved by taking acetaminophen although it relapsed after the medication wore off. He did not have any recent occurrences of bleeding conditions such as hematuria, epistaxis, hematochezia, or melena. In addition, he did not have any other significant past medical comorbidities alongside his current illness. The patient asserted that he did not recall any recent history of bites from animals or insects. He also had no specific habitual history including smoking, alcohol consumption, opium usage, or having any contact with animals or pets. He had not traveled in the past year except for the recent trip. In this regard, none of his fellow travelers showed similar symptoms.

His physical examination upon arrival was as follows: blood pressure of 87/60 mmHG, pulse rate of 80 beats per minute, respiratory rate of 17 breaths per minute, oxygen saturation of 98%, and fever of 39.1°C. He was conscious and alert, in a sound mental state, with pupils of moderate size that reacted appropriately to light. His hydration status was suboptimal. Both his skin turgor and tongue examination indicated mild dehydration. He showed no sign of jaundice in either his sclera or skin. Both his chest and abdominal examinations were unremarkable. No abnormalities, such as tenderness, distention, reduced bowel sounds, or enlargement of the liver or spleen, were noted in the abdominal assessment. His urine output was normal. There were no indications of edema, rashes, ecchymosis, petechiae, purpura, or any animal or insects bites on his body or extremities. There were also no signs of swelling, tenderness, or redness in his joints.

His laboratory findings in the emergency unit were as follows: The complete blood count (CBC) results revealed a hemoglobin level of 12.9 g/dL, a hematocrit of 39%, a white blood cell count of 2800/mm³ (with differential results specifying 55% neutrophils, 35% lymphocytes, 7% monocytes, and 3% eosinophils), a platelet count of 110,000/mm³, and an RDW of 12.5%. The electrolyte analysis revealed a sodium level of 129 mEq/L and a potassium level of 3.3 mEq/L. The blood glucose level was within the normal range. His liver function tests showed values within the normal range, including aspartate aminotransferase (AST) at 33 U/L, alanine aminotransferase (ALT) at 28 U/L, and alkaline phosphatase (ALP) at 169 U/L. Furthermore, there were no abnormalities detected in the levels of total or direct bilirubin. Renal function tests showed serum creatinine of 1.6 mg/dL, accompanied by normal results in the urine analysis. His coagulation assay showed normal results. His erythrocyte sedimentation rate (ESR) was recorded as 12, and his C-reactive protein (CRP) level was measured at 39. The patient was admitted to the hospital for further evaluation.

During admission, an electrocardiogram (ECG) and chest X-ray (CXR) were requested to assess the patient's condition, and the results were normal. Abdominal sonography revealed mild splenomegaly (144 × 46 mm) with a normal echo pattern, accompanied by a liver of normal span, absence of ascites, and normal kidney appearance. Based on the admission laboratory findings (low platelet count and high CRP), the patient was carefully assessed for multiple differential diagnoses, including probable infectious diseases.

Considering that the patient was referred during one of the significant peaks of COVID variants, specifically during the Omicron variant peak in Iran, a COVID-19 infection was suspected but ruled out following specific polymerase chain reaction (PCR) testing using commercial COVID-19 RT-PCR kit (Pishtaz Teb Diagnostics, Tehran, Iran). Furthermore, to rule out parasitic infections such as malaria, or abnormalities in platelet function such as thrombotic thrombocytopenic purpura (TTP) or immune thrombocytopenia (ITP), a peripheral blood smear was requested. However, no abnormalities were observed. The blood sample was also analyzed for hepatitis (using the GB SURASE B-96 (HBsAg), General Biologicals Corporation (GBC), Hsinchu County, Taiwan) and HIV (using the VIDAS HIV DUO Ultra, BioMérieux, Marcy l'Etoile, France), and the results were negative.

Given the patient's recent travel history to Indonesia, along with the presented symptoms and the absence of any other apparent underlying cause for persistent fever and thrombocytopenia, suspicion of dengue fever arose. Although petechiae and mosquito bites were not observed during the initial physical examination, the tourniquet (tie) test yielded a positive result, with more than 10 petechiae appearing below the antecubital fossa after the cuff was inflated. Consequently, the patient's blood was subjected to RT-PCR and enzyme-linked immunosorbent assay (ELISA) testing for dengue. The PCR test returned a positive result for dengue fever (using the QIAGEN OneStep RT-PCR Kit, QIAGEN GmbH, Hilden, Germany). Furthermore, both IgM and IgG tests for dengue antibodies yielded positive results in the patient's sample (using the Anti-Dengue Virus ELISA (IgG/IgM), EUROIMMUN, PerkinElmer, Lübeck, Germany). Serology results, evaluated semiquantitatively by calculating the ratio of the extinction of the patient sample to the calibrator, showed ratios of 17 for IgM and 13 for IgG, both significantly above the positive threshold ratio (1.1), indicating a typical acute dengue fever infection.

As supportive care and symptomatic therapy constitute the primary management strategy for dengue, the patient received meticulous nursing surveillance and intravenous fluid therapy with Lactated Ringer's solution, calculated based on his body weight of 97 kg. The fluid management included an initial bolus of 1500 mL administered over 1 hour, followed by a maintenance fluid regimen of 137 mL/hour (approximately 3288 mL/day), to address fluid loss and dehydration. Paracetamol and ondansetron were prescribed to alleviate fever, nausea, and vomiting. Daily CBC tests were requested to monitor platelet levels and evaluate for potential bleeding tendencies.  Table 1 outlines the chronological order of key clinical laboratory tests performed during the hospitalization. Recognizing the patient's ongoing gastrointestinal symptoms and considering the possibility of coinfections, empirical antibiotic therapy was initiated upon admission, comprising intravenous metronidazole and ceftriaxone.

Given the suspicion of coinfection with Salmonella or Brucella, both Widal and Wright agglutination tests were performed. The Wright test yielded negative results (using RITON BIO ANALYZE, Zist Gostaran Kosha Co. Ltd., Tehran, Iran). The Widal test showed a titer of 1:40 for Salmonella Paratyphi (OB antibodies) and 1:20 for Salmonella Typhi (OD antibodies). However, the patient was considered negative for salmonellosis, as a titer of 1:160 or higher is typically required for a positive diagnosis in endemic regions such as Iran [9, 10]. To further confirm these findings, blood cultures were obtained on two separate occasions, both yielding negative results. Despite these findings, antibiotic therapy was continued throughout hospitalization, following the previously established treatment regimen.

**Figure 1:**
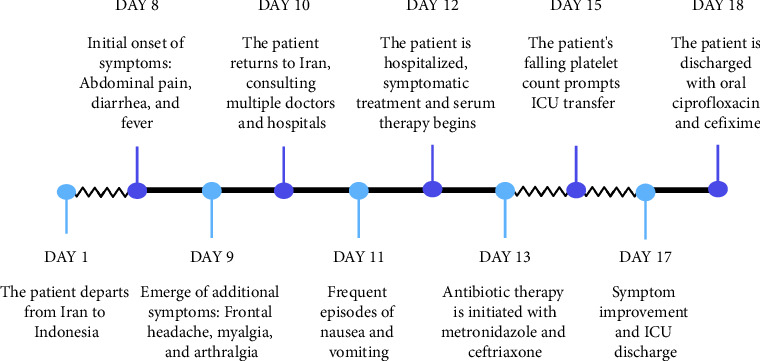
Patient's journey since arrival in Jakarta, highlighting key events and symptom progression.

As the decline in the platelet count and rise in the hematocrit levels in dengue fever may indicate an impending hemorrhage, and given that the patient's platelet count had decreased to 86,000/mm³ by the fourth day of admission, the decision was made to transfer him to the intensive care unit (ICU). Upon ICU admission, his condition was alarming, characterized by a preshock status with the possibility of progressing to full shock. His initial vital signs showed a heart rate of 115 beats per minute, blood pressure of 100/60 mmHg, and a respiratory rate of 22 breaths per minute. He was afebrile with a temperature of 36.8°C. Intravenous crystalloid fluids were administered to maintain adequate hydration, with fluid therapy carefully titrated to ensure a urine output of 0.5 cc/kg per hour. His hematocrit levels were monitored every 6 hours as an indicator of plasma leakage, rising to a peak of 44% two days after ICU admission. This, along with a further drop in platelet count to 35,000/mm³, necessitated continued intensive monitoring. Peripheral perfusion was carefully assessed by checking capillary refill time and peripheral pulses, with any abnormalities closely observed as potential signs of shock. Continuous hemodynamic monitoring was implemented, including noninvasive blood pressure measurement every two hours, pulse oximetry to ensure adequate oxygenation, with no observed decrease in oxygen saturation, and regular assessment of temperature and pulse rate, and monitoring of blood glucose levels. The patient's hypotension was compensated by fluid therapy, and his blood pressure stabilized without the need for vasopressors. The group and cross-matching were prepared for fresh packed cell transfusion due to the preshock state, but fortunately, transfusion was not needed. Electrolyte imbalances were corrected as needed, with particular attention to hypocalcemia, and daily arterial blood gas analysis was performed to assess acid–base balance, with particular attention to early signs of acidosis. Daily CBC examinations were conducted to track hematological trends, and the patient was examined daily for possible pleural effusion. In addition, the patient was monitored for early signs of shock and bleeding, including gastrointestinal bleeding, petechiae, and mucosal hemorrhages. Fortunately, no bleeding complications developed, and over the subsequent days, the thrombocytopenia resolved without any hemorrhagic events. The patient's symptoms gradually improved, and by the sixth day, his platelet count had increased to 48,000/mm³, showing an upward trend. He was discharged on the seventh day after admission with oral antibiotics, ciprofloxacin, and cefixime, as a precautionary measure due to a secondary bacterial infection. The timeline of events and an overview of symptoms from the onset of the patient's travel to Jakarta are illustrated in Figure 1. The patient was followed up at 2 weeks, 3 months, and 6 months postdischarge, and he remained afebrile and in good health with no further complications.

## 3. Discussion

Dengue fever, an acute febrile disease triggered by infection with the DENV, is an infectious cause of fever of unknown origin (FUO) [11]. DENVs are primarily transmitted from person to person through the bite of an infected Aedes mosquito, which also spreads chikungunya and Zika viruses. However, they can also be transmitted through blood transfusion, organ transplantation, and pregnancy [12].

Dengue is primarily endemic in tropical and subtropical regions, with sporadic cases reported in nonendemic areas due to travel, climate change, and other factors. The case presented here highlights the challenges associated with dengue fever diagnosis and management in a nonendemic country, particularly during the concurrent COVID-19 outbreak. Iran, as a nonendemic area for dengue fever, has previously experienced sporadic dengue cases. The first reported case of dengue in Iran was a 61-year-old man who had traveled to Kuala Lumpur, Malaysia, in 2008. He presented with symptoms including fever, fatigue, rash on his hands, and gum bleeding [8]. A subsequent Iranian case reported a 58-year-old woman in 2013 who experienced fever and bodily pain associated with skin rashes four days before admission. She had traveled to Malaysia 10 days prior to falling ill [13]. Another reported case in Iran in 2016 involved a 39-year-old woman who had symptoms similar to the common cold and a travel history to Malaysia. She exhibited bodily pain, lethargy, nausea, vomiting blood, ostealgia, and myalgia 3 days postreturn [14]. In the presented case, one of the foremost challenges was the initial misdiagnosis of dengue fever. The patient's symptoms, including fever and gastrointestinal manifestation, overlapped with various other febrile illnesses, leading to diagnostic confusion.

The simultaneous presentation of dengue fever in the presented case during a new COVID-19 subvariant wave (Omicron peak) in Iran further complicated the diagnostic process. While classic symptoms such as fever, headache, and myalgia were present, the pandemic status and the high patient load with similar COVID-19 presentations initially diverted attention from dengue fever as a differential diagnosis. During this peak of the COVID-19 pandemic in Iran, patients exhibited gastrointestinal symptoms along with fever [15]. Consequently, fever accompanied by typical gastrointestinal symptoms was often diagnosed as COVID-19, and if the patient was stable, they were advised to quarantine at home and informed about potential warning signs. Furthermore, bites from Aedes aegypti, the primary dengue carrier, can manifest as papules, which may have gone unnoticed due to overlapping clinical presentations. Given the challenges in differential diagnosis and patient management during a pandemic, it is crucial to involve experienced and vigilant medical specialists in emergency department triage areas to ensure thorough screening and prevent the oversight of other critical illnesses.

Furthermore, the patient's recent travel to an endemic region for dengue fever was not immediately recognized. Consequently, despite extensive diagnostic workup and evaluation for other infectious etiologies, dengue fever was not initially considered. Given Iran's close geographic proximity to endemic countries, along with increased international interactions such as trade and travel, the importation of new cases of dengue into Iran is plausible [16]. This case underscores the importance of not underestimating nonendemic diseases and infections, even if their occurrence is unlikely. Thus, in cases of febrile illnesses of unknown origin, it is essential to consider occupational and travel-related diseases that may result in uncommon infections in nonendemic regions. Therefore, a thorough social history including travel history and occupational exposure should be taken. In our case, the patient was referred to different centers and received ineffective treatments before it was realized that he had recently traveled to Jakarta. This oversight could have resulted in life-threatening conditions if not properly addressed and diagnosed.

Most people recover from dengue after about a week, but a person can be infected with dengue multiple times [17]. The management is centered on symptom relief, including rest, plenty of fluids, and acetaminophen. However, in some cases, dengue can progress to more severe forms, such as DHF. Petechiae may develop naturally or following slight trauma, such as with a positive tourniquet test, indicating capillary fragility and low platelet count [18]. Monitoring for petechiae and other hemorrhagic complications is essential, as they may suggest widespread infection and a potential progression to severe dengue. Severe dengue is a medical emergency, so timely identification and prudent administration of fluid therapy are crucial in preventing the onset of shock and mitigating its associated complications in patients. In the current case, the patient's clinical course, characterized by a progressive decline in platelet count and subsequent resolution without hemorrhagic complications, aligns with the typical trajectory of uncomplicated dengue fever. The decision to transfer the patient to the ICU upon detecting a declining pattern of thrombocytopenia exemplifies the proactive approach to manage potential complications associated with severe dengue, such as bleeding diathesis and plasma leakage syndrome.

Like other viral illnesses, dengue fever can be further complicated by concurrent infections with various viruses, bacteria, and Plasmodium species [19]. In this regard, enteric fever, caused by Salmonella Typhi, exhibits symptoms similar to dengue fever [20]. Concurrent dengue and typhoid fever infections have been reported previously [19–21]. In the mentioned case, although Salmonella coinfection was initially suspected based on the patient's symptoms, subsequent Widal test findings were inconclusive. The patient underwent antibiotic therapy with metronidazole and ceftriaxone from the second day of admission, and this regimen continued even after the negative blood culture result, which is the gold standard for identifying salmonellosis. This decision was based on diagnostic reliance on clinical manifestations rather than solely on laboratory tests in cases of salmonellosis, due to the potential for false positive and false negative results. Moreover, the patient displayed symptoms indicative of a potential coinfection concurrent with dengue, even if it was not confirmed as salmonellosis. This issue highlights the challenge of differentiating between dengue and other febrile infections solely based on clinical and laboratory parameters. Administering empirical antibiotic therapy in this case highlights the importance of considering concurrent infections in dengue cases, especially in patients with uncommon or persistent symptoms.

Dengue can be prevented by avoiding mosquito bites. Effective measures include using insect repellents, wearing clothing that covers the arms and legs, and using mosquito nets while traveling to tropical and subtropical warm and wet regions. Pretravel consultation before visiting an endemic area is another effective preventive measure for international travelers [22]. Moreover, a new dengue vaccine has been approved for use in children aged 6 through 16 years with laboratory-confirmed previous DENV infection and living in areas where dengue is endemic; however, it is not approved for travelers to areas where dengue is common [23]. In addition, advanced deep learning models have showed potential in accurately predicting dengue fever outbreaks up to 3 months in advance, which can help inform public health interventions and outbreak prevention strategies in endemic regions [24].

## 4. Conclusion

The presented scenario demonstrates the complexities of diagnosing dengue fever during concurrent outbreaks, such as COVID-19, where overlapping symptoms can lead to diagnostic confusion. Our findings underscore the necessity for healthcare providers to remain vigilant and consider a wide range of differential diagnoses, including uncommon infections in nonendemic regions. The complexity of managing dengue fever is further heightened by the potential for concurrent infections. In this context, to prevent life-threatening consequences of missed or delayed diagnoses, the initiation of empirical antibiotic therapy is a decision that specialists may consider on a case-by-case basis. In the current study, challenges regarding the correct diagnosis in patients with febrile illnesses of unknown origin also highlight the critical importance of patient history, particularly travel history and occupational exposures. In such cases, uncommon local infections, such as dengue fever, should not be disregarded.

## Figures and Tables

**Table 1:** Clinical laboratory findings during hospitalization.

Test description (unit)	Days of admission							Reference range
	1st day	2nd day	3rd day	4th day	5th day	6th day	7th day	
*Complete blood counts*								
WBC (×10^3^/μL)	2.8	2.4	2.2	2	3.9	5	6.2	4–10
Hb (g/dL)	12.9	12.4	13.2	14.1	13.9	14.8	14	14–18
Hct (%)	39.4	36.5	38.2	41.6	40.3	44.1	40	42–52
Plt (×10^3^/μL)	110	97	90	86	35	48	75	150–450
RDW (%)	12.5	42.4	39.6	40.4	40.9	41.5	39.8	40–53

*Biochemical analysis*								
Cr (mg/dL)	1.6	1.5	1.52	1.5	1.72	1.38	1.2	0.7–1.4
CRP (mg/L)	39	—	—	7.5	—	2.9	—	Up to 6

Abbreviations: CRP, C-reactive protein; Cr, creatinine; Hb, hemoglobin; Hct, hematocrit; Plt, platelet count; WBC, white blood cells.

## Data Availability

The data that support the findings of this study are available from the corresponding author upon reasonable request.

## Nomenclature

ALP: Alkaline phosphatase
ALT: Alanine aminotransferase
AST: Aspartate aminotransferase
CBC: Complete blood count
CRP: C-reactive protein
CXR: Chest X-ray
DENV: Dengue virus
DHF: Dengue hemorrhagic fever
DSS: Dengue shock syndrome
ECG: Electrocardiogram
ELISA: Enzyme-linked immunosorbent assay
ESR: Erythrocyte sedimentation rate
FUO: Fever of unknown origin
ICU: Intensive care unit
ITP: Immune thrombocytopenia
PCR: Polymerase chain reaction
TTP: Thrombotic thrombocytopenic purpura
